# Risk Assessment after ST-Segment Elevation Myocardial Infarction: Can Biomarkers Improve the Performance of Clinical Variables?

**DOI:** 10.3390/jcm11051266

**Published:** 2022-02-25

**Authors:** Alvaro Garcia-Osuna, Jordi Sans-Rosello, Andreu Ferrero-Gregori, Aitor Alquezar-Arbe, Alessandro Sionis, Jordi Ordóñez-Llanos

**Affiliations:** 1 Department of Clinical Biochemistry, Hospital de la Santa Creu i Sant Pau, 08041 Barcelona, Spain; 2 Department of Clinical Biochemistry, Autonomous University of Barcelona, 08193 Barcelona, Spain; 3 Department of Cardiology, Hospital de la Santa Creu i Sant Pau, 08041 Barcelona, Spain; 4 Department of Emergency, Hospital de la Santa Creu i Sant Pau, 08041 Barcelona, Spain

**Keywords:** cardiovascular, STEMI, risk assessment, biomarkers, multibiomarker, clinical model, NT-proBNP, GDF-15, CT-IGFBP-4

## Abstract

Introduction: Myocardial infarction with ST-segment elevation (STEMI) is the coronary artery disease associated with the highest risk of morbimortality; however, this risk is heterogeneous, usually being evaluated by clinical scores. Risk assessment is a key factor in personalized clinical management of patients with this disease. Aim: The aim of this study was to assess whether some new cardiac biomarkers considered alone, combined in a multibiomarker model or in association with clinical variables, improve the short- and long-term risk stratification of STEMI patients. Materials and Methods: This was a retrospective observational study of 253 patients with STEMI. Blood samples were obtained before or during the angiography. The assessed biomarkers were C-terminal fragment of insulin-like growth factor binding protein-4 (CT-IGFBP4), high sensitive cardiac troponin T (hs-cTnT), N-terminal fragment of probrain natriuretic peptide (NT-proBNP), and growth differentiation factor 15 (GDF-15); they reflect different cardiovascular (CV) physiopathological pathways and underlying pathologies. We registered in-hospital and follow-up mortalities and their causes (cardiovascular and all-cause) and major adverse cardiac events (MACE) during a two year follow-up. Discrimination, survival analysis, model calibration, and reclassification of the biomarkers were comprehensively evaluated. Results and Discussion: In total, 55 patients (21.7%) died, 33 in-hospital and 22 during the follow-up, most of them (69.1%) from CV causes; 37 MACE occurred during follow-up. Biomarkers showed good prognostic ability to predict mortality, alone and combined with the multibiomarker model. A predictive clinical model based on age, Killip–Kimball class, estimated glomerular filtration rate (eGFR), and heart rate was derived by multivariate analysis. GDF-15 and NT-proBNP significantly improved risk assessment of the clinical model, as shown by discrimination, calibration, and reclassification of all the end-points except for all-cause mortality. The combination of NT-proBNP and hs-cTnT improved CV mortality prediction. Conclusions: GDF-15 and NT-proBNP added value to the usual risk assessment of STEMI patients.

## 1. Introduction

Cardiovascular diseases are the leading cause of death globally, causing around one-third of all registered deaths [1]. Atherosclerosis is one of the causes more frequently related to cardiovascular diseases in general and to coronary heart disease in particular. Acute myocardial infarction (AMI) is one of the most life-threatening complications of atherosclerosis. Two types of AMI can be differentiated on the basis of the ECG tracing: ST-segment elevation myocardial infarction (STEMI) and non-ST-segment elevation myocardial infarction (NSTEMI). Currently, depending on the age of the population, it is estimated that 25–40% of AMI are STEMI [2,3]. Percutaneous coronary intervention (PCI) is the gold-standard treatment for STEMI. Coronarography permits one to evaluate the extent of coronary lesions [4] and identify the culprit lesion when multivessel disease is found [5]. However, despite being the most adequate therapy, the rate of in-hospital and post-discharge complications in STEMI is high and heterogeneous, related not only to the extension of the myocardial injury but also to other factors such as age, culprit vessel size and flow, the presence of comorbidities [6,7], or the existence of atheroma plaques prone to rupture [8]. Apart from coronarography lesions and the result of their treatment, the assessment of short- and long-term risk of STEMI patients relies on biomarkers of myocardial injury and clinical scores such as GRACE 2.0 (Global Registry of Acute Coronary Event) [9]. Some studies have shown that biomarkers other than those of cardiac injury could improve risk prognosis in STEMI patients [10,11]. Of these, heart failure (HF) biomarkers are among the more common and B-type natriuretic peptides (BNPs) have an established role in risk assessment of MI patients [12]. Other biomarkers could also be helpful in refining the risk assessment in STEMI signaling processes before or during the MI. Pregnancy-associated plasma protein-A (PAPP-A) is a metalloproteinase synthesized by the syncytiotrophoblast during pregnancy, but also, among other cells, by monocytes and macrophages [13]. PAPP-A was suggested to be an early biomarker of acute coronary syndromes (ACS) when it was found in eroded atherosclerotic plaques, but not in stable ones [14,15]. Later, some studies demonstrated that PAPP-A induces a pro-thrombotic phenotype in endothelial cells by inducing expression of functionally active tissue factor [16] and that transcoronary PAPP-A levels are significantly higher in ACS patients as compared to patients with stable coronary artery disease, indicating that PAPP-A plays a role in the pathophysiology of ACS [17]. Unfortunately, the potential of PAPP-A as a biomarker in ACS cannot be fully translated into clinical practice in patients treated with unfractioned heparin, since this drug, widely used in the ACS context, promotes an acute release of PAPP-A from cell surface to the circulation [18]. PAPP-A proteolyzes the insulin-like growth factor binding protein 4 (IGFBP-4) that, in turn, unbinds the insulin growth factor-1 (IGF-1), promoting low-density cholesterol uptake, macrophage activation, and proinflammatory cytokines release [13,19]. Consequently, IGFBP-4 fragments reflect the PAPP-A activity in the IGF-1/IGFBP-4 atherosclerotic pathway and are insensitive to heparin’s action [20]. IGFBP-4 fragments (amino- and carboxi-terminal) have demonstrated a capacity to predict complications in patients with symptoms of myocardial ischemia [21] and association with increased all-cause and cardiovascular mortality rates in type 1 diabetes patients [22]. Growth differentiation factor 15 (GDF-15) is a transforming growth factor beta superfamily (TGFβ) cytokine secreted by macrophages and cardiomyocytes in response to oxidative stress and inflammation. It has been confirmed that GDF-15 is expressed in atheroma plaques located on coronary arteries [23]. The relationship between GDF-15 concentration and cardiovascular risk factors [24], and other biomarkers such as C-reactive protein (CRP), cardiac troponin (cTn), or BNPs has been established. In STEMI, GDF-15 concentrations are slightly increased after the episode [25] and are suggested to identify patients at high risk of adverse left ventricular remodeling and hospitalization for HF after an ACS [26]. Different studies have demonstrated that high concentrations of GDF-15 are associated with an increased risk of mortality in patients with ACS [27]. GDF-15 has been also proposed as a biomarker of frailty, a state of functional decline, which increases the vulnerability of patients to suffering adverse effects and worsens the prognosis of all cardiovascular diseases, including STEMI [28]. In this context, we propose that risk assessment in STEMI patients could be improved by recently developed cardiac biomarkers considered alone or combined with cTn and BNPs or analyzed together with clinical variables.

## 2. Material and Methods

### 2.1. Study Design and Patients

This retrospective observational study included patients with STEMI, older than 18 years and recruited between January 2013 and November 2014, as a sub-study of a national registry (Red de Investigación Cardiovascular (RIC)) of ACS at one university hospital. Patients referred from other hospitals where they had undergone PCI and those without coronary occlusion in the angiography were excluded. Demographic and anthropometric variables, severity scores, and infarction-related characteristics were registered. Patients were followed up for 2 years after discharge by appointments or phone calls, or through electronic health records. The assessed outcomes were death from all or cardiovascular causes, both in-hospital and in follow-up, and major adverse cardiac events (MACE) defined as the composite of the need of additional PCI or coronary artery bypass graft (CABG), reinfarction, or cardiovascular death. Cardiovascular death included death by cardiogenic shock, heart failure, arrhythmic storm, or myocardial rupture.

### 2.2. Laboratory Variables

All blood samples were obtained within the first 30 min after the patient’s arrival at the hospital (in the emergency department or in the catheterization laboratory). Most samples were obtained before the catheterization, but depending on the clinical workout (e.g., patients with previous arrest or in poor cardiac condition), some samples were obtained from peripheral veins a few minutes after the cardiologists had begun the catheterization. After centrifugation (15 min, 4500× *g*), plasma aliquots were frozen at −80 °C until analysis. Long-term stability of the study biomarkers at −80 °C have shown negligible or minimum decreases in their immunoreactivity, otherwise not affecting their clinical meaning [22,29,30]. Routine biochemical and hematological measurements were determined at baseline. Regarding study biomarkers, we measured in microtiter plates the CT-IGFBP-4 concentrations by a research-use-only enzyme-linked immunosorbent assay (ELISA) provided by Mercodia AB (Uppsala, Sweden). The ELISA uses a sandwich of two monoclonal antibodies directed against separate antigenic determinants of the molecule. A 5-point-calibration curve (4.9–180 µg/L) was run in each plaque. Validation parameters of the research version assay were not provided by the manufacturer; however, in a recent paper, within- and total-assay imprecision of <6.0% and <9.7%, respectively, were reported [31]. Amino-terminal portion of the probrain natriuretic peptide B (NT-proBNP), GDF-15, and high sensitivity cTnT (hs-cTnT) were measured by electrochemiluminescent immunoassays (Roche Diagnostics, Basel, Switzerland). According to the manufacturer, the limit of detection (LoD) of the NT-proBNP assay is 5 ng/L, and the maximum detectable concentration is 35,000 ng/L. The intra- and inter-assay imprecisions were <2.4% and <2.6%, respectively, in a concentration range between 130 and 4942 ng/L. In patients with suspected acute HF, a determination of NT-proBNP < 300 ng/L provides a very high negative predictive value of the condition [32]. LoD of the GDF-15 assay is 400 ng/L, and the maximum reportable concentration is 20,000 ng/L. The intra- and inter-assay imprecisions were <0.9% and <2.3%, respectively, in a concentration range between 1100 and 18,600 ng/L. Different decision values had been previously proposed [26]. Regarding hs-cTnT assay, a measuring range of 5–10,000 ng/L is reported by the manufacturer. Intra- and inter-assay imprecisions were <3.6% and <1.9%, respectively, in a range of 27.2–2278 ng/L. Myocardial injury is considered to exist when the hs-cTnT concentration exceeds 14 ng/L, the upper reference limit of the assay [33].

### 2.3. Statistical Analysis

The evaluation of the different biomarkers was performed following the most up to date recommendations [34,35]. Continuous variables were expressed as median and interquartile range (IQR) and were compared using the Mann–Whitney *U* test. Categorical variables were expressed as absolute values and percentage, and were compared using the χ^2^ test. The role of biomarkers in discrimination of patients with and without outcomes was assessed in several ways. First, by receiver operating characteristic (ROC) curves; the univariate areas under the curve (AUC) were compared with a multibiomarker model, including the four biomarkers by DeLong’s method. The best prognostic cutoff values obtained in the ROC analysis were used in Kaplan–Meier survival analysis, and incidence distributions in patients with and without the outcomes were compared using the log-rank test. Survival analyses were also performed using Cox proportional hazards models. A model integrating biomarkers and clinical variables to assess patients’ risk was derived by lineal regression. The power of the biomarkers to increase outcome prediction was evaluated by calibration (Hosmer–Lemeshow test, Brier Score (BS), and Akaike Information Criterion (AIC)), discrimination (Integrated Discrimination Index (IDI)), and reclassification analyses. Category-free net reclassification index (NRI) was calculated as the sum of the “event NRI” (NRI_e_) and the “nonevent NRI” (NRI_ne_); upwards movements in patients meeting an outcome implied improved classification, whereas downward movements implied worse reclassification. Statistical analysis was performed with the statistical packages SPSS 24.0 (IBM Corp. Released 2016. IBM SPSS Statistics for Windows, Version 24.0 IBM Corp. Armonk, NY, USA) and R (www.R-project.org).

## 3. Results

### 3.1. Basal Variables

**Figure 1:**
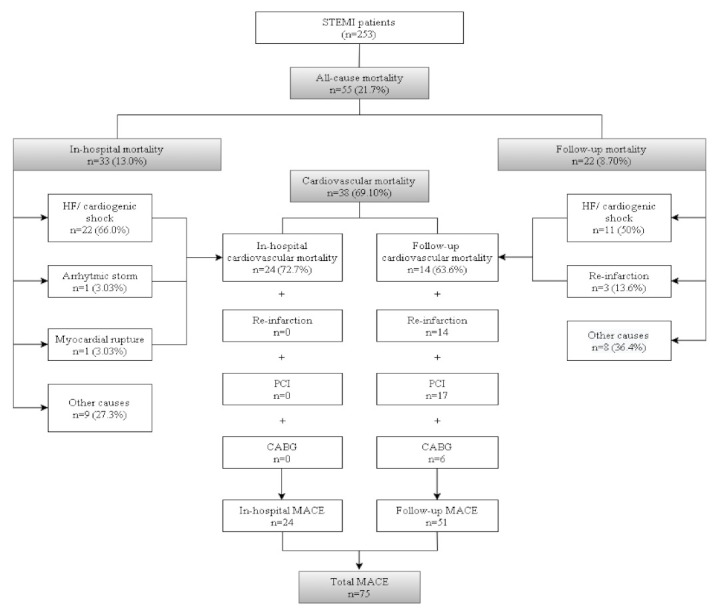
Diagram describing all the events registered during the 2 years of follow-up. The assessed outcomes are marked in gray. Abbreviations: CABG: coronary artery bypass graft, HF: heart failure, MACE: major adverse cardiac events, PCI: percutaneous coronary intervention.

The study included 253 STEMI patients, 66 (26.1%) of whom were women who were older than the men (median age 75 vs. 63 years; *p* < 0.01). No other relevant differences were observed between genders. Characteristics of the patients and patient’s infarction and the results of laboratory variables are summarized in Table 1 (and extensively detailed in Table S1). In our cohort, door-to-balloon time was <30 min in 85.6% of these patients. Median (IQR) ischemia times (time from onset of symptoms-balloon) were 183 (157–315) min. Among the 253 patients included, emergent coronary angiography was performed in 249 patients (98.4%). There were four patients (1.58%) in whom coronary angiography was not performed due to previous high comorbidity and/or deteriorated vital status. Among patients who underwent coronary angiography, two patients (0.79%) died during the procedure. PCI was performed in 94.9% of cases, and successful coronary reflow (TIMI flow 3–4) was obtained in 93.8% of patients. Regarding outcomes, 33 patients (13.0%) died in-hospital, and 22 (8.7%) more during the two year follow-up (median follow-up of 912 days), yielding an overall mortality of 21.7% (Figure 1). No patients were lost to follow-up. All patients were discharged with double antiplatelet therapy: aspirin 220/220 (100%), second anti-platelet agent: clopidogrel 146/220 (66.36%), prasugrel 51/220 (23.18%), ticagrelor 22/220 (10.0%); angiotensin-converting enzyme inhibitors/angiotensin II receptor blockers (ACRI/ARB) 162/12 (78.63%), aldosterone receptor antagonists (MRA) 47/220 (21.36%), cardioselective beta-blockers 162/220 (73.63%) and statins 218/220 (99.1%). A total of 17/220 (7.72%) were also discharged with anticoagulant therapy (acenocumarol).

Two-thirds (69.1%) of deaths were due to cardiovascular causes. After the two-year follow-up, we registered 75 MACE (24 in-hospital, 51 after discharge). Patients who died during hospitalization presented more frequently high-risk criteria such as higher Killip class—93.3% of patients with Killip class IV died during the study—and GRACE 2.0 score, lower LVEF, hypotension, multivessel disease, and cardiac arrest at presentation (data not shown). Regarding laboratory variables, patients who died in-hospital had significantly higher concentrations of glucose and creatinine, higher AST and ALT activities, and more leukocytosis. Table S1 shows detailed demographic and clinical characteristics of the patients and compares such characteristics between patients with and without the outcomes.

### 3.2. Cardiovascular Biomarkers

Median concentrations of the studied biomarkers are shown in Table 2. Unsurprisingly, median hs-cTnT concentrations were extremely high, corresponding to extensive myocardial damage caused by the STEMI; CT-IGFB4 and GDF-15 median values were 1.5 to 2 times higher than the associated to lower frequency or severity of the assessed outcomes. Finally, although median NT-proBNP values were below described cutoff values, median concentrations of patients meeting any of the outcomes were above such cutoffs (Table S1).

When comparing the variables between the patients with and without the outcome, we found that patients with outcomes were older than those without, except for MACE. Higher Killip–Kimball class and GRACE 2.0 score values, higher number of affected vessels and revascularization success, higher glycemia, worse renal function, and increased concentrations of CV biomarkers were significantly more frequent in patients with outcomes in all outcome subgroups (Table S1).

### 3.3. Risk Assessment

The discrimination ability of the CV biomarkers to predict outcomes was assessed for each single biomarker by the AUC of the ROC curve and compared against a multibiomarker model including all the biomarkers (Table 3). The multibiomarker model improved the AUC of the single biomarkers to predict mortality except for (a) GDF-15 for predicting in-hospital and all-cause mortality, and (b) NT-proBNP for predicting cardiovascular mortality. In contrast, the multibiomarker model did not improve the capacity of prediction of MACE of any single biomarker.

The best prognostic cutoff values to predict outcomes were calculated by ROC analysis. We found that a concentration of 63.0 µg/L for CT-IGFBP-4, 988 ng/L for hs-cTnT, and 1454 ng/L for NT-proBNP were predictive of all outcomes, except mortality in the follow-up that was predicted by a slightly lower value. GDF-15 cutoff values ranged between 2724 and 3124 ng/L, depending on the outcome (Table S2). These cutoff values were applied to a survival analysis using the log-rank test of the Kaplan–Meier survival analysis (detailed in Table S3) and a Cox proportional hazard model analysis. All biomarkers were predictors of outcomes in a univariate analysis. However, when adjusted for the GRACE 2.0 score, the better association was found between GDF-15 and in-hospital (Hazard ratio (HR) of 14.28) and all-cause (HR 4.60) mortalities and for NT-proBNP and cardiovascular mortality (HR 4.05); MACE was predicted by hs-cTnT (HR 2.18) and NT-proBNP (HR 2.32) (Table 4).

In order to integrate biomarkers and the clinical variables used in daily practice, we calculated a lineal regression model. All candidate variables were incorporated into a statistical model if they had showed statistical differences (*p* < 0.05) for the different outcomes (Table S1). The final clinical model included age, Killip–Kimball class, estimated glomerular filtration rate (eGFR by CKD-EPI), and heart rate. To evaluate the incremental usefulness of CT-IGFBP-4, hs-cTnT, NT-proBNP, and GDF-15 over the clinical model, we conducted discrimination, calibration, and reclassification tests. Non-normally distributed variables were log-transformed prior to these analyses. Results of the discriminating capacity (as AUC and IDI) are shown in Table S4.

Discrimination results, especially AUC comparison, showed that although the information provided by some biomarkers was valuable, they provided a small addition to the robust baseline clinical model. According to the IDI results, the discriminating capacity of the clinical model’s value improved by adding GDF-15 (2.97%) for in-hospital mortality, NT-proBNP+hs-cTnT (5.58%) for cardiovascular mortality, and hs-cTnT (2.02%) for MACE; all-cause and follow-up mortality discrimination was not improved by any biomarker used alone or in combination.

To evaluate the correlation between the predictions of the different models and the observed outcomes, we performed a series of calibration tests (Table S5). The AIC and Brier Score (BS) were calculated for each model; lower values indicate a better model. According to the results, the best-calibrated model for in-hospital mortality was the one that included the clinical model and GDF-15 (BS 0.067; AIC 119). The clinical model plus hs-cTnT and NT-proBNP was the most accurate at predicting cardiovascular mortality (BS 0.073; AIC 135). Clinical model and NT-proBNP showed good performance for MACE (BS 0.157; AIC 262). Regarding reclassification of patients, the addition of the multibiomarker strategy to the clinical model reclassified patients in all the events except follow-up mortality. For in-hospital mortality, the addition of GDF-15 and the multibiomarker strategy improved the overall prediction (NRI 55.8% and 67.3%, respectively), particularly improving the ability to predict events (NRI_e_ 39.4% and 45.45%, respectively); the model including GDF-15 also improved all-cause mortality prediction (NRI: 35.5%; NRI_e_: 27.3%). For cardiovascular mortality, the addition of NT-proBNP, hs-cTnT, or both also allowed us to reclassify patients (NRI 56.5%, 69.2%, or 74.8%; NRI_e_ 42.1%, 47.4%, and 47.4%, respectively). Finally, only the combined clinical and multibiomarker model produced a significant reclassification (NRI 43.8%), again with a predominance of event reclassification (NRI_e_ 30.2%) in patients with MACE (Table 5).

Taking into account all the obtained results, we summarize the best strategies for predicting outcomes in STEMI and the evidence supporting them in Table 6.

## 4. Discussion

Mortality associated with STEMI is high, around 5–6% during hospital admission and up to 18% at 6 months after discharge [37]. STEMI mortality is heterogeneous and difficult to predict because it is multifactorial; clinical factors such as advanced age, the extent of the infarcted area, or the existence of comorbidities increase the average mortality. In the present group, we registered a mortality rate of 21.7% after a 2 year follow-up; most of them (13%) occurred during hospitalization. Cardiovascular were the most frequent cause of death (69% of all deaths) during the whole observational study. The observed mortality was high compared to that reported in other studies in STEMI patients. This could have been due to two reasons: First, our hospital is a referral center for patients with STEMI initially diagnosed at other health centers; second, the population that attends our hospital is older than in similar centers and has a higher frequency of age-associated risk factors or comorbidities. This justifies our study being enriched with a high proportion of STEMI patients with severe conditions—as shown by a Killip–Kimball class IV presented by 25.7% of patients, a median GRACE 2.0 risk score of 179, or higher frequency of cardiac arrest at presentation (8.30%) or atrial fibrillation (5.5%). In this context, the elevated in-hospital mortality rate is not surprising. The largest Spanish registry of ACS (MASCARA registry) observed an in-hospital mortality rate in STEMI patients of 5.7% [38], more than two times lower than that observed in the present group. In our population, 93.9% of patients who died during admission were Killip IV; the mortality of these patients was as high as 47.7%. High mortality associated with Killip IV was already described in the original article that originated this classification [39] and has been corroborated in several studies, such as the CardShock study in patients with cardiogenic shock, in which mortality rose up to a 40% [40]. The high risk profile of our population is also corroborated by the fact that one-third of patients developed a major adverse cardiac event.

Median concentrations of NT-proBNP and GDF-15 were much higher in patients who had any of the studied outcomes. The capacity of GDF-15 to predict in-hospital mortality, all-cause mortality, and MACE was not improved by a model that included all the assessed biomarkers; the same was observed for the prediction of NT-proBNP and cardiovascular mortality. In a multivariate analysis adjusted for the GRACE 2.0 score, NT-proBNP and GDF-15 concentrations above the calculated cut offs continued to be associated with a higher risk of in-hospital death, specifically, 2.3-fold for NT-proBNP and 14.3-fold for GDF-15, and with death in the follow-up (NT-proBNP 6.95-fold). Regarding cardiovascular mortality, the biomarker that individually best predicted the outcome was NT-proBNP (AUC 0.809, 95%CI 0.731–0.887). For MACE prediction, the four assessed biomarkers presented a similar predictive capacity, but were clearly lower (AUC between 0.608 and 0.661) than those found for any type of mortality. Evaluation of association and discrimination capacity is helpful for the evaluation of new biomarkers prior to their use in clinics, but it is not enough. Biomarker values and clinical scores should be combined with calibration and reclassification studies to improve their clinical potential. A typical example of such combination is the GRACE 2.0 score, validated for risk assessment in many clinical conditions including STEMI [41]. However, the score includes only a qualitative evaluation of cardiac biomarkers—positive/negative—instead of a quantitative result obtained with the best biomarker and technology available (i.e., a cardiac troponin measured with a high-sensitivity method). Given the lack of optimization of the GRACE 2.0 score, we derived a clinical model in our population that finally included age, Killip–Kimball class, eGFR-CKD-EPI, and heart rate. The clinical model showed excellent discrimination for in-hospital, cardiovascular, and all-cause mortality with AUC > 0.890 (>0.700 for MACE). The good performance of the clinical model is not surprising because it includes powerful morbidity and mortality predictors such as age, Killip class, and renal function status. In fact, age is the only variable of the model that is also included in all the scores used for risk stratification in STEMI: GRACE 2.0, TIMI (Thrombolysis in Myocardial Infarction) and dynamic TIMI, CADILLAC (Controlled Abciximab and Device Investigation to Lower Late Angioplasty Complications), Zwolle, and PAMI (Primary Angioplasty in Myocardial Infarction) [42].

Deviation between observed and predicted risk was assessed using different calibration methods. When biomarkers were added to the clinical model, we observed that GDF-15 and the combination of hs-cTnT and NT-proBNP significantly improved the prediction of in-hospital and cardiovascular mortality of the clinical model, respectively, whereas MACE prediction was ameliorated by NT-proBNP. Biomarkers did not improve prediction of follow-up and all-cause mortality compared to the clinical model. Given the predominance of cardiovascular deaths in our population, the ability of biomarkers to predict all-cause mortality and MACE could be decreased by the low number of noncardiovascular outcomes. If we analyze the capacity of biomarkers to improve prognosis by the increase they promote on the AUC of the clinical model, any of them improve its discrimination for any outcome. The fact that the predictive capacity of the clinical model was so high prevented biomarkers from improving their discrimination [43]. However, using IDI, we found that some strategies did improve outcome prediction. It has been stated that the IDI is a better alternative to AUC comparison that overcomes its limitations and could be considered a more powerful statistical test [44]. Regarding reclassification, we calculated continuous NRI to allow comparisons with other studies. Otherwise, when risk categories are used, the NRI result can be influenced by the arbitrary choice of risk levels. It has also been stated that the use of reclassification strategies with poorly calibrated models can lead to misinterpretation of the results [45]. This situation was observed in two of the outcomes assessed (follow-up and all-cause mortality) where poor calibrated models, shown by a non-reduction in Brier Score and AIC after adding the biomarkers, produced false reclassification proportions. On the other hand, for in-hospital and cardiovascular mortality, the addition of biomarkers to the clinical model showed a significant reclassification of patients. Pencina et al. [43] suggested interpretations of the continuous NRI: NRI < 20% is weak, NRI 20–40% is intermediate, and NRI > 60% is considered strong. Continuous NRI in the present study ranged between 29.0% and 74.8%. Most patients reclassified on the basis of NRI were reclassified as “event”, indicating that they were at a higher and missed risk by the clinical model alone. The identification of such patients will be helpful in improving their clinical management. Different studies have found an association between IGFBP-4 fragments and an increased risk of all-cause mortality, cardiovascular mortality, and MACE in patients with STEMI [46], as well as with all-cause mortality in patients with AHF [31] after adjustment for clinical variables and other biomarkers such as NT-proBNP and CRP. In our cohort, on the basis of the whole statistical analysis results, we cannot confirm these findings. We found that after adjustment for clinical variables, the only biomarker not associated with an improvement of the risk prediction was CT-IGFBP-4. In the last 10 years, several studies suggest that the most useful tools to predict MACE are imaging techniques, such as cardiovascular magnetic resonance or cardiac computed tomography angiography [47,48].

## 5. Conclusions

In a population of STEMI patients treated according to recommendations and through measuring for the first time a combination of four biomarkers (CT-IGFBP-4, hs-cTnT, NT-proBNP, and GDF-15) with more recent and automated technology available, we found that:

− The STEMI patient group was enriched with critical patients and comorbid patients compared to other studies. This circumstance provides great support to the biomarkers’ prognostic value and could be highlighted as a strength of the study.
− A determination of GDF-15 on admission improved the prognostic capacity of in-hospital mortality compared to a model based only on clinical variables. It could help to grade the treatment required by patients.
− The combination of NT-proBNP and hs-cTnT improved the ability of a powerful clinical model to predict cardiovascular mortality.
− CT-IGFBP-4, proposed as a biomarker of unstable atheroma plaques implicated in STEMI, was not associated with a significant improvement of the risk models with clinical variables and other biomarkers.

## 6. Limitations

Given the composition of the study group, enriched with older and critical patients compared to other groups, our results should be corroborated in different STEMI populations. Some blood samples from our study were obtained a few minutes after the cardiologist had begun the catheterization. Reflow of the obstructed coronary artery leads to an abrupt release of biomarkers into the circulation; this fact may represent a confounding factor compared to studies that obtained the samples prior to PCI. However, previous publications have demonstrated that biomarkers obtained after PCI are as useful for risk assessment as those measured in prePCI samples [49,50,51,52]. Even though patients were recruited several years ago, the reference population and characteristics of our hospital has not changed; our role in the local STEMI network is the same, and thus the spectrum of patients is nowadays similar to the time of recruitment. Furthermore, the guidelines for the treatment of these patients and the internal protocols have not significantly changed either. In regard to the applicability of the studied biomarkers, both hs-cTnT and NT-proBNP are well-introduced in routine clinical practice. In contrast, GDF-15 has been analyzed in many clinical situations, but its measurement is not recommended in guidelines for clinical practice; moreover, its cost is usually higher than that of hs-cTnT or NT-proBNP. Finally, there is no CT-IGFBP-4 commercial assay available, and only some manufacturers offer specific antibodies to develop in-house measurement methods. The number of patients included was low. The findings should be corroborated in more extensive cohorts.

## Figures and Tables

**Table 1:** Baseline characteristics of patients and their myocardial infarction (STEMI) and routine laboratory results.

Demography and Antecedents (*n* = 253, Unless Otherwise Indicated)			
Patients and STEMI Characteristics		Laboratory Analysis	
Sex, woman	66 (26.1)	Sodium (mmol/L)	138 (136–140)
Age (years)	66 (55–76)	Potassium (mmol/L)	3.80 (3.43–4.10)
BMI (kg/m^2^)	26.1 (24.9–29.1)	Glucose (mmol/L)	8.90 (7.10–12.60)
Hypertension	157 (62.1)	Urea (mmol/L)	7.00 (5.00–9.00)
Dyslipidemia	124 (49.0)	Creatinine (µmol/L)	82.0 (69.0–106)
Smoking status	110 (43.5)	eGFR (CKD-EPI; mL/min/1.73 m^2^)	81.5 (62.5–90)
Type 2 diabetes	55 (21.7)	AST (U/L)	63.0 (28.0–168)
Killip–Kimball class ^1^		CK (U/L)	290 (139–804)
I	125 (49.4)	Total cholesterol (mmol/L)	4.34 (3.59–5.09)
II	42 (16.6)	HDL cholesterol (mmol/L)	0.97 (0.82–1.15)
III	21 (8.30)	LDL cholesterol (mmol/L)	2.66 (2.02–3.39)
IV	65 (25.7)	Hemoglobin (g/L)	136 (123–152)
Cardiac arrest	21 (8.30)	Hematocrit (L/L)	40.0 (36.0–44.0)
GRACE 2.0 Risk Score ^1^	179 (145–232)	Platelets (×10^9^/L)	212 (169–252)
Ischemia time (min)	208 (160–320)	Leukocytes (×10^9^/L)	11.9 (9.42–14.8)
Systolic BP (mm Hg)	115 (85–135)	HbA1c (%)	5.70 (5.40–6.10)
Diastolic BP (mm Hg)	70 (53–80)		
LVEF %	44 (35–55)		
PCI	240 (94.9)		

Data presented as number of cases (% occurrence) or median (interquartile range). ^1^ Killip–Kimball class and GRACE risk score are variables evaluating the vital risk of an individual patient; Killip–Kimball III and IV are associated with worse, even fatal outcomes, and a GRACE score > 150 is associated with a >12% mortality in the 6 month period following an acute MI. Abbreviations: BMI: body mass index, PCI: percutaneous coronary intervention, GRACE 2.0: Global Registry of Acute Coronary Events, BP: blood pressure, LVEF: left ventricular ejection fraction, eGFR CKD-EPI: estimated glomerular filtration rate by the Chronic Kidney Disease Epidemiology Collaboration formula, AST: aspartate amino transferase, CK: creatine kinase, HDL and LDL: high- and low-density lipoproteins, HbA1c: glycated hemoglobin A1c.

**Table 2:** Biomarkers of the study.

Biomarker	Total Group Median (IQR) (*n* = 253)	Deceased Patients Median (IQR) (*n* = 55)	Surviving Patients Median (IQR) (*n* = 198)	Reference Values
CT-IGFBP-4, µg/L	48.7 (32.9–67.8)	69.9 (42.6–91.1)	45.0 (30.1–60.5)	Not evaluated in the literature; a concentration < 34.3 µg/L is associated with the lowest mortality and MACE occurrence in STEMI patients [20]
hs-cTnT, ng/L	373 (84.0–1614)	1148 (146–3868)	351 (80–1209)	A concentration > 14 ng/L indicates myocardial injury
NT-proBNP, ng/L	508 (99.0–1897)	2201 (519–14,004)	329 (84–1197)	Depending on the patient’s age < 300 or <900 or <1800 ng/L rules-out acute heart failure
GDF-15, ng/L	2297 (1328–5323)	6989 (3215–20,000)	1918 (1187–3496)	A concentration > 1200 ng/L is associated to incresed mortality in STEMI patients [36]

**Table 3:** Discrimination capacity—AUC of ROC curves of CT-IGFBP-4, hs-cTnT, NT-proBNP, GDF-15, and multibiomarker strategy—for predicting different outcomes.

Biomarker	In-Hospital Mortality		Follow-Up Mortality		Cardiovascular Mortality		All-Cause Mortality		MACE	
	AUC (95%CI)	*p*-Value	AUC (95%CI)	*p*-Value	AUC (95%CI)	*p*-Value	AUC (95%CI)	*p*-Value	AUC (95%CI)	*p*-Value
CT-IGFBP-4	0.656	<0.001	0.765 (0.658–0.872)	0.002	0.731	<0.001	0.713	<0.001	0.625	0.379
	(0.539–0.772)				(0.640–0.822)		(0.628–0.798)		(0.541–0.709)	
hs-cTnT	0.632	<0.001	0.610 (0.488–0.732)	<0.001	0.697	<0.001	0.611	<0.001	0.608	0.215
	(0.531–0.732)				(0.609–0.785)		(0.528–0.695)		(0.528–0.688)	
NT-proBNP	0.716	0.003	0.746 (0.632–0.861)	0.001	0.809	0.138	0.740	0.002	0.661	0.791
	(0.612–0.820)				(0.731–0.887)		(0.660–0.819)		(0.579–0.743)	
GDF-15	0.874	0.457	0.717 (0.604–0.829)	0.005	0.808	0.042	0.819	0.130	0.616	0.055
	(0.802–0.946)				(0.727–0.889)		(0.749–0.888)		(0.528–0.704)	
Multibiomarker	0.877	Ref	0.810 (0.722–0.899)	Ref	0.865	Ref	0.848	Ref	0.668	Ref
	(0.809–0.946)				(0.806–0.924)		(0.790–0.907)		(0.583–0.754)	

Abbreviations: 95%CI: 95% confidence interval; *p*-value of the comparison (DeLong’s test) with the multibiomarker strategy; Ref: reference model for comparison.

**Table 4:** Kaplan–Meier analysis and Cox regression survival analysis (univariate and adjusted for GRACE 2.0.) of CT-IGFBP-4, hs-cTnT, NT-proBNP, and GDF-15 for the different outcomes using the predefined cut-off values.

Biomarker	In-Hospital Mortality			Follow-Up Mortality			Cardiovascular Mortality		
	Log-Rank χ^2^ (*p*-Value)	Univariate HR (95%CI)	Adjusted for GRACE 2.0 HR (95%CI)	Log-Rank χ^2^ (*p*-Value)	Univariate HR (95%CI)	Adjusted for GRACE 2.0 HR (95%CI)	Log-Rank χ^2^ (*p*-Value)	Univariate HR (95%CI)	Adjusted for GRACE 2.0 HR (95%CI)
CT-IGFBP-4	14.33 (<0.001)	3.48 (1.74–6.94)	NS	23.56 (<0.001)	6.80 (2.77–16.69)	4.85 (1.92–12.23)	23.99 (<0.001)	4.48 (2.31–8.66)	2.46 (1.26–4.81)
hs-cTnT	7.33 (0.007)	2.48 (1.25–4.93)	NS	4.48 (0.034)	NS	NS	20.92 (<0.001)	4.20 (2.14–8.21)	3.21 (1.63–6.30)
NT-proBNP	22.90 (<0.001)	4.77 (2.34–9.70)	2.30 (1.13–4.70)	14.41 (<0.001)	9.83 (2.30–42.06)	6.95 (1.60–30.30)	40.71 (<0.001)	7.07 (3.50–14.27)	4.05 (1.99–8.24)
GDF-15	61.67 (<0.001)	55.02 (7.51–402.8)	14.28 (1.84–110.88)	16.39 (<0.001)	5.58 (2.19–14.27)	3.44 (1.16–10.17)	38.01 (<0.001)	10.64 (4.10–27.26)	3.65 (1.28–10.38)
**Biomarker**	**All-Cause Mortality**			**MACE**					
	**Log-Rank χ^2^** **(*p*-Value)**	**Univariate HR (95%CI)**	**Adjusted for GRACE 2.0 HR (95%CI)**	**Log-Rank χ^2^** **(*p*-Value)**	**Univariate HR (95%CI)**	**Adjusted for GRACE 2.0 HR (95%CI)**			
CT-IGFBP-4	35.43 (<0.001)	4.50 (2.61–7.77)	2.49 (1.43–4.33)	23.99 (<0.001)	1.86 (1.12–3.08)	NS			
hs-cTnT	9.43 (0.002)	2.24 (1.32–3.81)	NS	9.43 (0.002)	2.41 (1.47–3.96)	2.18 (1.32–3.58)			
NT-proBNP	35.74 (<0.001)	4.49 (2.61–7.01)	2.41 (1.39–4.19)	35.74 (<0.001)	2.91 (1.77–4.76)	2.32 (1.38–3.90)			
GDF-15	62.72 (<0.001)	12.22 (5.52–27.04)	4.60 (1.93–10.99)	62.72 (<0.001)	2.51 (1.51–4.16)	NS			

Abbreviations: 95%CI: 95% confidence interval; HR: hazard ratio; NS: not significant (*p* > 0.05).

**Table 5:** Reclassification (Net Reclassification Index) of the clinical model plus the addition of the log-transformed concentrations of CT-IGFBP-4, hs-cTnT, NT-proBNP, and GDF-15 for the different outcomes.

	In-Hospital Mortality			Follow-Up Mortality			Cardiovascular Mortality		
	NRI_e_ % (*p*-Value)	NRI_ne_ % (*p*-Value)	NRI % (*p*-Value)	NRI_e_ % (*p*-Value)	NRI_ne_ % (*p*-Value)	NRI % (*p*-Value)	NRI_e_ % (*p*-Value)	NRI_ne_ % (*p*-Value)	NRI % (*p*-Value)
CM + log CT-IGFBP-4	21.21 (NS)	5.45 (NS)	26.67 (NS)	0 (NS)	15.2 (0.020)	15.2 (NS)	−10.53 (NS)	6.05 (NS)	−4.48 (NS)
CM + log hs-cTnT	9.09 (NS)	0 (NS)	9.09 (NS)	9.09 (NS)	1.3 (NS)	10.39 (NS)	26.30 (NS)	19.19 (0.004)	45.40 (0.008)
CM + log NT-proBNP	21.21 (NS)	−7.27 (NS)	13.94 (NS)	0 (NS)	3.9 (NS)	3.9 (NS)	42.10 (0.004)	14.40 (0.032)	56.50 (<0.001)
CM + log hs-cTnT + log NT-proBNP	-	-	-	-	-	-	47.40 (<0.001)	21,90 (<0.001)	69.20 (<0.001)
CM + log GDF-15	39.40 (0.014)	16.40 (0.014)	55.80 (0.001)	−9.09 (NS)	3.9 (NS)	−5.19 (NS)	21.05 (NS)	0.465 (NS)	21.52 (NS)
CM + Multibiomarker	45.45 (0.003)	21.80 (<0.001)	67.30 (<0.001)	9.09 (NS)	10.82 (NS)	19.91 (NS)	47.40 (<0.001)	27.40 (<0.001)	74.80 (<0.001)
	**All-Cause Mortality**			**MACE**					
	**NRI_e_ %** **(p-value)**	**NRI_ne_ % ** **(*p*-Value)**	**NRI %** **(*p*-Value)**	**NRI_e_ %** **(*p*-Value)**	**NRI_ne_ % ** **(*p*-Value)**	**NRI %** **(*p*-Value)**			
CM + log CT-IGFBP-4	−5.45 (NS)	2.02 (NS)	−3.43 (NS)	7.94 (NS)	6.32 (NS)	14.25 (NS)			
CM + log hs-cTnT	9.09 (NS)	6.06 (NS)	15.15 (NS)	14.29 (NS)	7.37 (NS)	21.65 (NS)			
CM + log NT-proBNP	20.0 (NS)	6.06 (NS)	26.06 (NS)	2.02 (0.022)	14.70 (0.040)	29.00 (0.043)			
CM + log GDF-15	27.27 (0.035)	8.08 (NS)	35.35 (0.017)	−1.59 (NS)	1.05 (NS)	−0.54 (NS)			
CM + Multibiomarker	9.09 (NS)	16.16 (0.020)	25.25 (NS)	30.2 (0.012)	13.70 (0.050)	43.80 (0.001)			

Abbreviations: CM: clinical model; NRI: Net Reclassification Index; NRI_e_: event NRI; NRI_ne_: nonevent NRI; NS: not significant (*p* > 0.05).

**Table 6:** Best risk assessment strategy for each analyzed outcome and its statistical analysis evidence.

	In-Hospital Mortality	Follow-Up Mortality	Cardiovascular Mortality	All-Cause Mortality	MACE
Best risk assessment strategy	Clinical model ^1^ + GDF-15	Clinical model	Clinical model + hs-cTnT + NT-proBNP	Clinical model	Clinical model + NT-proBNP
Evidence	Discrimination (IDI) Survival analysis (HR) Calibration (Brier Score, AIC) Reclassification (NRI_e_/NRI_ne_)		Discrimination (IDI) Calibration (Brier Score, AIC) Reclassification (NRI_e_/NRI_ne_)		Survival analysis (HR) Calibration (Brier Score, AIC) Reclassification (NRI_ne_)

^1^ Clinical model based on age, Killip–Kimball class, eGFR by CKD-EPI and heart rate. Abbreviations: AIC: Akaike Information Criterion; HR: hazard ratio; IDI: Integrated Discrimination Index; NRI: Net Reclassification Index; NRI_e_: event NRI; NRI_ne_: nonevent NRI.

## Supplementary Materials

The following supporting information can be downloaded at https://www.mdpi.com/article/10.3390/jcm11051266/s1, Table S1: Baseline demographics and clinical characteristics as a function of in-hospital mortality, follow-up mortality, cardiovascular mortality, all-cause mortality, and MACE. Table S2: Cut-offs values of CT-IGFBP-4, hs-cTnT, NT-proBNP, and GDF-15 for the different outcomes. Table S3: Kaplan–Meier survival analysis with survival rates per subgroups. Table S4: Discrimination (AUC and IDI) of the clinical model plus the addition of the log-transformed concentrations of CT-IGFBP-4, hs-cTnT, NT-proBNP, and GDF-15 for the different outcomes. Table S5: Calibration (Hosmer–Lemeshow test, Brier Score, and AIC) of the clinical model plus the addition of the log-transformed concentrations of CT-IGFBP-4, hs-cTnT, NT-proBNP, and GDF-15 for the different outcomes.
